# Preoperative Pregabalin and the Cp50 for Skin Incision During Target-Controlled Propofol Infusion: A Randomized, Placebo-Controlled, Double-Blind Clinical Trial

**DOI:** 10.1213/ANE.0000000000007824

**Published:** 2025-10-29

**Authors:** Johannes Müller, Roxana Polojintef-Corbu, Stefan Ulbing, Alexandra Graf, Birgit Reiter, Thomas Stimpfl, Walter Plöchl

**Affiliations:** From the 1 Clinical Division of General Anaesthesia and Intensive Care Medicine, Department of Anaesthesia, Intensive Care Medicine and Pain Medicine; 2 Institute for Medical Statistics, Center for Medical Statistics, Informatics and Intelligent Systems; 3 Department of Laboratory Medicine, Medical University of Vienna, Vienna, Austria.

## Abstract

**BACKGROUND:**

The potency of propofol is measured by its Cp50 value, which is the plasma concentration required to suppress a motor response in 50% of patients during surgical incisions. This Cp50 value can be affected by the concurrent use of other drugs, including opioids, benzodiazepines, or lidocaine. Pregabalin, a medication commonly administered as preoperative premedication, provides mild sedative and anxiolytic effects. Although pregabalin has the potential to reduce the minimum alveolar concentration (MAC) of sevoflurane, its effects on propofol-based anesthesia have not been conclusively studied. Thus, we designed a placebo-controlled, double-blind clinical trial to evaluate the impact of pregabalin on the Cp50 of propofol.

**METHODS:**

Eighty female patients who underwent breast surgery participated in this study. They received either a placebo or 300 mg of pregabalin 2 hours before anesthesia induction. Propofol was administered as the sole anesthetic agent, delivered continuously via a target-controlled infusion (TCI) pump using the Schnider model, without the addition of opioids or other analgesics. Patients in both groups were administered different target effect-site propofol concentrations, and their motor responses to standardized skin incisions were determined. The Cp50 value of propofol was estimated using a logistic regression model, and the results were re-evaluated using bootstrap methods.

**RESULTS:**

A significant difference in propofol Cp50 values was found between the placebo and pregabalin groups. Using the delta method, the Cp50 value of propofol was estimated to be 16.9 μg/mL (95% confidence interval [CI], 15.1–18.8) in the placebo group and 9.4 μg/mL (95% CI, 4.46–14.3) in the pregabalin group. Secondary outcome measures revealed significantly decreased opioid consumption and pain levels in the recovery area in the pregabalin group compared to the placebo group.

**CONCLUSIONS:**

This study demonstrated that pretreatment with 300 mg pregabalin significantly reduced the Cp50 value of propofol by 44%, as calculated using the delta method. When 300 mg of pregabalin is administered before anesthesia and propofol is used for maintenance via TCI, a lower target effect-site concentration might be sufficient. Additionally, pregabalin premedication has the potential to decrease postoperative pain and opioid consumption.

**KEY POINTS**

- **Question:** Does preoperative administration of pregabalin reduce the Cp50 value of propofol during target-controlled infusion anesthesia for surgical skin incision?
- **Findings:** Preoperative pregabalin administration significantly decreased the Cp50 of propofol by 34%, indicating a deeper anesthesia level with a lower propofol concentration.
- **Meaning:** Pregabalin premedication may enhance anesthetic depth and reduce postoperative pain and opioid use, potentially improving anesthetic management in surgical patients.

Pregabalin is an anticonvulsant drug used perioperatively to manage neuropathic pain and anxiety disorders. It is classified not as a gamma-aminobutyric acid (GABA) receptor agonist but as an agent targeting the α-2-delta subunit of voltage-dependent calcium channels.^1^ It binds to this specific site, modulates calcium influx at the nerve terminals, and inhibits the release of excitatory neurotransmitters.^1^ This depresses the central nervous system, resulting in sedation, anxiolysis, and modulation of pain perception.^2^

Therefore, pregabalin has been increasingly used in the perioperative period to reduce anxiety and manage pain.^3^ Given its growing use, understanding the interactions between pregabalin and other common anesthetic agents is essential. We recently demonstrated that perioperative pregabalin administration reduced sevoflurane’s minimum alveolar concentration (MAC).^4^ However, its interaction with propofol, one of the most important anesthetic agents, remains inadequately studied.

The potency of propofol is commonly quantified by its Cp50 value, the plasma concentration required to prevent a movement response in 50% of patients during surgical incisions. Various drugs, such as opioids and benzodiazepines, have been shown to influence the Cp50 value of propofol.^5^

Currently, there is scientific debate about the effects of pregabalin on the Cp50 of propofol, with studies reporting either no effect or synergistic effects based on EEG parameters.^6,7^ To provide more definitive evidence, evaluating a more concrete outcome parameter may clarify these conflicting results. We hypothesize that synergistic effects can be observed if such parameters are used and the Cp50 of propofol be reduced if pregabalin is used perioperatively.

**Table 1.** Subject Characteristics, Secondary Outcome Parameters, and Side Effects in the Placebo and the Pregabalin Groups

Parameter	Placebo (n = 40)	Pregabalin (n = 40)	*P* Value
Age (y)	50.8 (10.3)	49.0 (11.6)	.477
BMI (kg/m^2^)	23.2 (2.58)	24.8 (3.00)	.0152
ASA physical status (n)			
I	28	30	.617
II	12	10	
Equilibration time (min)	21.6 (2.45)	22.7 (7.69)	.370
Duration of surgery (min)	60.3 (24.7)	60.4 (41.2)	.992
Body temperature at incision (°C)	36.0 (0.31)	36.0 (0.29)	.707
Plasma pregabalin concentration (μg/mL)	0 (0)	7.67 (3.00)	NA
Piritramide intraoperative (mg)	3.49 (0.98)	3.34 (1.38)	.560
BIS before incision	7 (8.22)	10 (10.4)	.395
BIS after incision	7 (6.63)	11 (9.83)	.135
Systolic blood pressure before incision (mm Hg)	97 (16.5)	97 (13.4)	.809
Systolic blood pressure after incision (mm Hg)	103 (14.1)	99 (14.0)	.249
Diastolic blood pressure before incision (mmHg)	56 (11.4)	55 (6.60)	.640
Diastolic blood pressure after incision (mm Hg)	61 (9.11)	61 (10.9)	.799
Heart rate before incision (beats/min)	71 (11.0)	66 (11.3)	.0425
Heart rate after incision (beats/min)	68 (13.3)	65 (14.5)	.251
Cumulative phenylephrine dose intra OP (mg)	0.495 (0.44)	0.504 (0.36)	.631
Piritramide intraoperative + PACU (mg/kg BW)	0.13 (0.07)	0.08 (0.04)	.0006
Piritramide in PACU (mg)	4.51 (3.91)	2.51 (2.64)	.0173
Patients receiving metamizole in the PACU (n)	10	9	.793
Patients receiving ondansetron in the PACU (n)	1	0	>.999
Postoperative pain score (NRS)	3.1 (1.77)	1.7 (1.77)	.0016
Overall satisfaction (1–4)	3.9 (0.27)	3.9 (0.22)	>.999
Anesthesia induction satisfaction (1–4)	3.7 (0.48)	3.7 (0.45)	.63
Total negative side effects (n)	4	7	.4541
Awareness (n)	0	0	NA
Nausea (n)	1	1	>.999
Vomiting (n)	0	0	NA
Dizziness (n)	3	6	.481
Visual impairment (n)	0	0	NA
Others (n)	0	0	NA

Data are presented as mean (standard deviation); satisfaction is coded as follows: 4 = very satisfied, 3 = somewhat satisfied, 2 = somewhat unsatisfied, 1 = not satisfied; NRS asks patients to give a number between 0 and 10 with 10 meaning the most intense imaginable pain and 0 no pain at all. BIS, blood pressure, and heart rate were measured 2 min before and after incision. Data are rounded to three significant digits.

Abbreviations: ASA, American Society of Anesthesiologists; BIS, bispectral index; BMI, body mass index; NRS, numeric rating scale for pain; OP, operative; PACU, postanesthesia care unit.

Therefore, we propose measuring the Cp50 of propofol by assessing motor response to a standardized painful stimulus, analogous to the MAC definition for volatile anesthetics. We designed and conducted a randomized, double-blind, placebo-controlled clinical trial to investigate the effect of pregabalin on the Cp50 of propofol. Additionally, we evaluated postoperative pain levels and opioid consumption.

## METHODS

### Ethics

This study was conducted according to the Consolidated Standards of Reporting Trials (CONSORT) guidelines and the Declaration of Helsinki. Approval was obtained from the local ethics committee (Ethics Committee of the Medical University of Vienna) and the regulatory authority (Bundesamt für Sicherheit im Gesundheitswesen) before patient enrollment commenced. The trial was registered before patient enrollment in EudraCT (registration number: 2021-003664-28; Principal Investigator: Walter Plöchl; date of registration: August 5, 2022). Patients were contacted at least 1 day before the planned operation, and written informed consent was obtained from all participants before enrollment in the trial.

### Study Design and Population

This prospective, randomized, controlled, single-center, double-blind clinical trial was conducted between December 2022 and November 2023. Patient enrollment began on December 20, 2022, and ended on November 19, 2023. The final follow-up was completed on November 20, 2023. We recruited only adult patients with an American Society of Anesthesiologists (ASA) physical status of I or II who were scheduled for elective surgery. For standardization, we included only female patients who underwent breast surgery. Furthermore, patients were required to be between 25 and 65 years old with a body mass index (BMI) between 18.5 and 30 (Table 1). Exclusion criteria included patients who could not understand the study procedures, required sedative or analgesic medication, had allergies to the study medications, or were unsuitable for laryngeal mask airway management.

### Randomization and Blinding

Patients were randomly assigned to receive either 300 mg of pregabalin (Pregabalin Ratiopharm, TEVA B.V.) or a placebo (maltodextrin) 1 to 2 hours before surgery. Randomization was performed using block randomization via the Randomization Service for Multicenter Clinical Trials (Institute for Medical Informatics, Statistics, and Documentation, Medical University of Graz). No stratification was performed. Patients were also assigned a patient-specific target concentration of propofol for induction and maintenance of anesthesia. A motor pump (Alaris PK Plus; Becton Dickinson Austria GmbH) was programmed according to the Schnider algorithm for propofol effect-site concentrations.^8^ All randomizations were performed in the operating theater by a study nurse who was not involved in assessing the motor response to skin incisions. Patients, surgeons, and postoperative care providers were blinded to group allocation.

The Vienna General Hospital pharmacy provided the pregabalin and placebo capsules. The investigators who induced anesthesia were blinded to the randomization results.

### Induction of Anesthesia

On entering the operating theater, patients underwent pulse oximetry, electrocardiography, noninvasive blood pressure monitoring, and bispectral index (BIS) monitoring. For anesthesia induction, only propofol (Propofol 1% MCT Fresenius; Fresenius Kabi Austria GmbH) was administered using the Schnider model,^8^ set to the effect-site concentration mode with the assigned patient-specific target concentration. The Schnider model was selected for its ability to account for patient-specific factors such as body weight, height, and age. Furthermore, it is widely used in both clinical research and routine practice, and allows for targeting specific effect-site concentrations.^8^

After starting the propofol infusion, the patients were ventilated with a facial ventilation mask for 4 to 6 minutes. When BIS values were <55, and patients were unarousable, a laryngeal mask airway (LMA^®^ Supreme Airway™,^*a*^ Teleflex Medical Europe Ltd, Dublin, Ireland) was inserted, and patients were mechanically ventilated to maintain end-tidal CO_2_ (etCO_2_) concentrations between 30 and 40 mm Hg. Forced-air warming was applied to maintain normothermia. An arterial line was placed to collect blood samples to determine propofol and pregabalin plasma concentrations.

### Determination of Cp50 of Propofol

After anesthesia induction, propofol continued to be the sole anesthetic agent and was continuously administered via a motor pump. The surgical site was then cleaned and prepared. Propofol infusion was maintained for at least 20 minutes to reach equilibrium between the effect-site and plasma. Subsequently, the surgeon made a standardized skin incision of 3 to 5 cm, depending on the exact location of the incision and type of operation. Two blinded investigators observed patients’ motor responses to the skin incision for 1 minute. One investigator assessed the head and upper extremities, while the other observed the lower extremities. If either investigator reported gross purposeful movement of the head or at least one extremity within 1 minute, the motor response was classified as “positive.” Coughing, bucking, and straining were not considered.^9^ If there was no movement, it was considered a negative response. Additional parameters such as heart rate, blood pressure, BIS index, and etCO_2_ were monitored.

### Propofol Target Effect-Site Concentrations and Sample Size Calculation

Simulation studies were conducted to determine the sample size for this study. The assumptions for these simulation studies were based on 3 published studies: Andrews et al,^10^ Weber et al,^5^ and Kazama et al.^11^ The underlying dose–response curves for propofol were based on logistic models for Andrews et al^10^ with *b*0 = −6.7 and *b*1 = 0.47 and, therefore, a Cp50 of 14.3 μg/mL; for Weber et al^5^ with *b*0 = −8.5 and *b*1 = 1 and therefore a Cp50 of 8.5 μg/mL and for Kazama et al^11^ with *b*0 = −3.8 and *b*1 = 0.38 and therefore a Cp50 of 10 μg/mL. Furthermore, due to clinical experience, it was decided not to administer target effect-site concentrations smaller than 5.5 μg/mL as intraoperative awareness is possible below this concentration.^12^ As the most plausible assumption for the dose–response curve of the placebo group, it was decided to use a logistic model with *b*0 = −10 and *b*1 = 1 and, therefore, a Cp50 of 10 μg/mL and a probability of no reaction to the stimulus of 1% at a propofol dose of 5 μg/mL.

Based on these assumptions, the targeted propofol concentrations were set to 5.5, 7, 8.5, 10, 11.5, 13, and 14.5 μg/mL. The simulation studies were conducted to estimate the statistical power of several sample size allocations under these assumptions. In each simulation step, data were randomly generated using the assumed dose–response curves and the Cp50 was estimated separately for the 2 groups using a logistic regression model for movement with propofol concentration as an independent factor. The corresponding 95% confidence interval (CI) was estimated using the delta method.^13^ Results in each simulation step were considered significant if the CIs did not overlap. We conducted 10,000 simulation runs per simulation scenario (ie, for each evaluated sample size allocation). The power in each simulation scenario was calculated as the proportion of significant results (nonoverlapping CIs) of the 10,000 simulation runs. Sample size allocation ratios were evaluated to achieve a power of at least 80%. The final allocation ratio was chosen from the allocation ratios achieving at least 80% power based on feasibility and to avoid underrepresentation of dosages at both ends of the dose–response curve.

In the placebo group, 4 patients were randomized to a propofol target effect-site concentration of 5.5 μg/mL, 5 patients to 7 μg/mL, 7 patients to 8.5 μg/mL, 8 patients to 10 μg/mL, 7 patients to 11.5 μg/mL, 5 patients to 13 μg/mL, and 4 patients to 14.5 μg/mL.

In the pregabalin group, 7 patients were randomized to a propofol target effect-site concentration of 5.5 μg/mL, 8 patients to 7 μg/mL, 7 patients to 8.5 μg/mL, 5 patients to 10 μg/mL, 5 patients to 11.5 μg/mL, 4 patients to 13 μg/mL, and 4 patients to 14.5 μg/mL.

The reported allocation matched the a priori sample size calculation and was fixed during the planning phase of the trial without adjustments throughout the study.

### Maintenance of General Anesthesia

After assessing the response to the skin incision, remifentanil was continuously administered at 0.1 to 0.3 µg/kg/min to facilitate the surgical procedure. Subsequently, propofol target effect-site concentration was reduced to 2 to 3 μg/mL to maintain steady anesthetic conditions. Phenylephrine was continuously administered as needed to maintain adequate blood pressure. Approximately 20 minutes before the end of the surgical procedure, 1000 mg of metamizole was administered for initial postoperative analgesia. Additionally, 1.5 to 4.5 mg piritramide was administered at the anesthesiologists’ discretion, considering factors such as body weight, body size, and physiological parameters, including blood pressure and heart rate. The clinicians were blinded to the randomization results for pregabalin or placebo premedication. All patients received 4 mg of dexamethasone to prevent nausea.

### Determination of Propofol and Pregabalin Plasma Concentrations

An arterial line was used to collect blood samples to determine propofol and pregabalin plasma levels. Blood samples for measuring pregabalin plasma concentrations were collected immediately before skin incision. Blood samples for determining propofol concentrations were drawn exactly 15 and 20 minutes after anesthesia induction. The samples were then transported to the Department of Laboratory Medicine at the Medical University of Vienna.

Pregabalin plasma concentrations were measured using liquid chromatography-tandem mass spectrometry (LC-MS/MS) with the IVD-CE certified Assay MassTox^®^ TDM (Chromsystems Instruments & Chemicals GmbH).

To determine propofol plasma concentrations, samples were centrifuged exactly 30 minutes after blood collection and then frozen at −20 °C until analysis. For propofol analysis, 0.05 mL of plasma was extracted with *n*-butyl chloride and analyzed by gas chromatography-tandem mass spectrometry (GC-MS/MS). The analytical method was validated for a range of 1 to 40 µg/mL plasma according to the European Medical Agency guideline on bioanalytical method validation (EMEA/CHMP/EWP/192217/2009).

### Pain Management, Recovery Room, and Postoperative Follow-Up

After the surgical procedure, the patients were transferred to the recovery room, where patient-controlled analgesia (PCA) was administered. Patients were asked to rank their pain levels according to the numeric rating scale for pain (NRS), which ranged from 0 (no pain at all) to 10 (most extreme imaginable pain). If their score was higher than 5, a single dose of 1000 mg metamizole and 3 mg piritramide was administered. Subsequently, bolus doses of 3.0 to 4.5 mg of piritramide were administered if the pain levels were higher than 2.

After 2 hours, patients were interviewed regarding any awareness during surgery or discomfort and adverse events such as nausea, dizziness, or visual impairment. Furthermore, the patients’ satisfaction level with anesthesia induction and overall anesthesia care were recorded. A scale reaching from 1 to 4 was used, with 4 meaning “very satisfied,” 3 “somewhat satisfied,” 2 “somewhat dissatisfied,” and 1 “not satisfied.” Patients were also asked to rate their pain levels according to their NRS.

### Statistical Analysis

A binary outcome was observed for every concentration tested in the placebo and pregabalin groups: either movement or no movement. Within each group, Cp50 was calculated using a logistic regression model for the binary outcomes (movement or no movement), with concentration as an independent factor. The corresponding 95% CIs were calculated using the delta method. Furthermore, the 95% CI for the difference of Cp50 values for the 2 independent groups (pregabalin, placebo) was calculated using the delta method. The difference was considered statistically significant, if the 95% CI of the Cp50 difference between groups did not include 0. As a sensitivity analysis, Cp50 and the corresponding 95% CIs were re-evaluated separately for both groups as well as for the difference between both groups using the bootstrap method.^13^ The bootstrap estimates for the Cp50 separately for both groups as well as for the difference between groups and the corresponding CIs were based on 10,000 resamples from the observed data set. Within each resample, the Cp50 was calculated separately for both groups using a logistic regression model for the binary outcome (movement/no movement) with concentration as an independent factor. Furthermore, the difference between the Cp50 of both groups was calculated. For the overall bootstrap estimates, the mean value and the 2.5% and 97.5% percentiles of the 10,000 resample estimates were calculated.^13^

**Table 2.** Cp50 of Propofol Plasma Concentrations

Cp 50 of propofol plasma concentrations		
Group	Delta method	Bootstrap
Placebo	16.9 (15.1–18.8)	17.0 (14.1–26.7)
Pregabalin	9.4 (4.46–14.3)	9.78 (4.33–16.6)
Difference	7.53 (0.39–14.7)	6.96 (−2.91 to 18.9)

Cp50 (µg/mL) and corresponding 95% confidence limits separately for the plasma concentrations of propofol in the placebo and the pregabalin group as well as for the difference between groups. Data are rounded to three significant digits.

**Figure 1.**
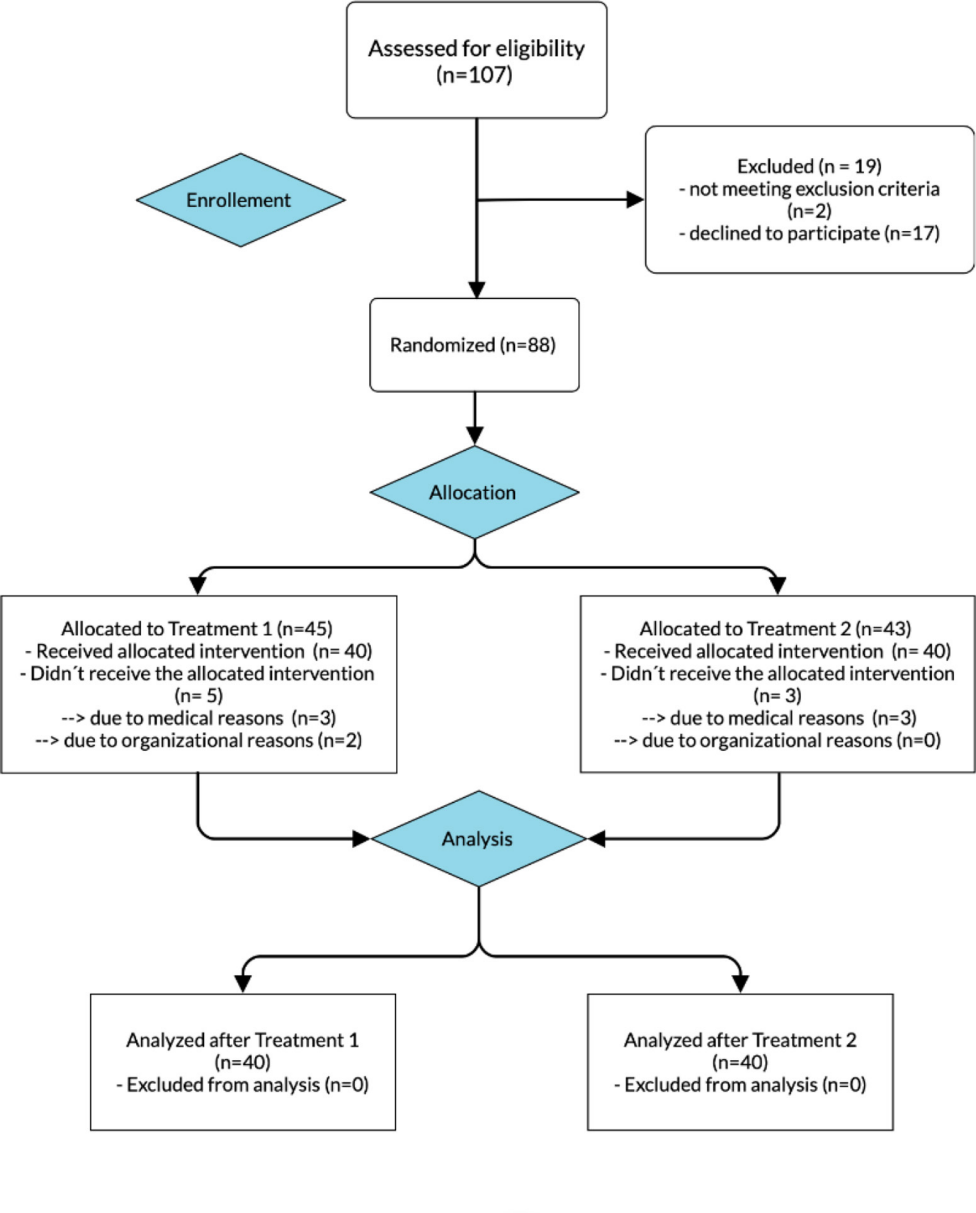
Consent flowchart detailing the enrollment, allocation, and analysis phases of the study.

**Figure 2.**
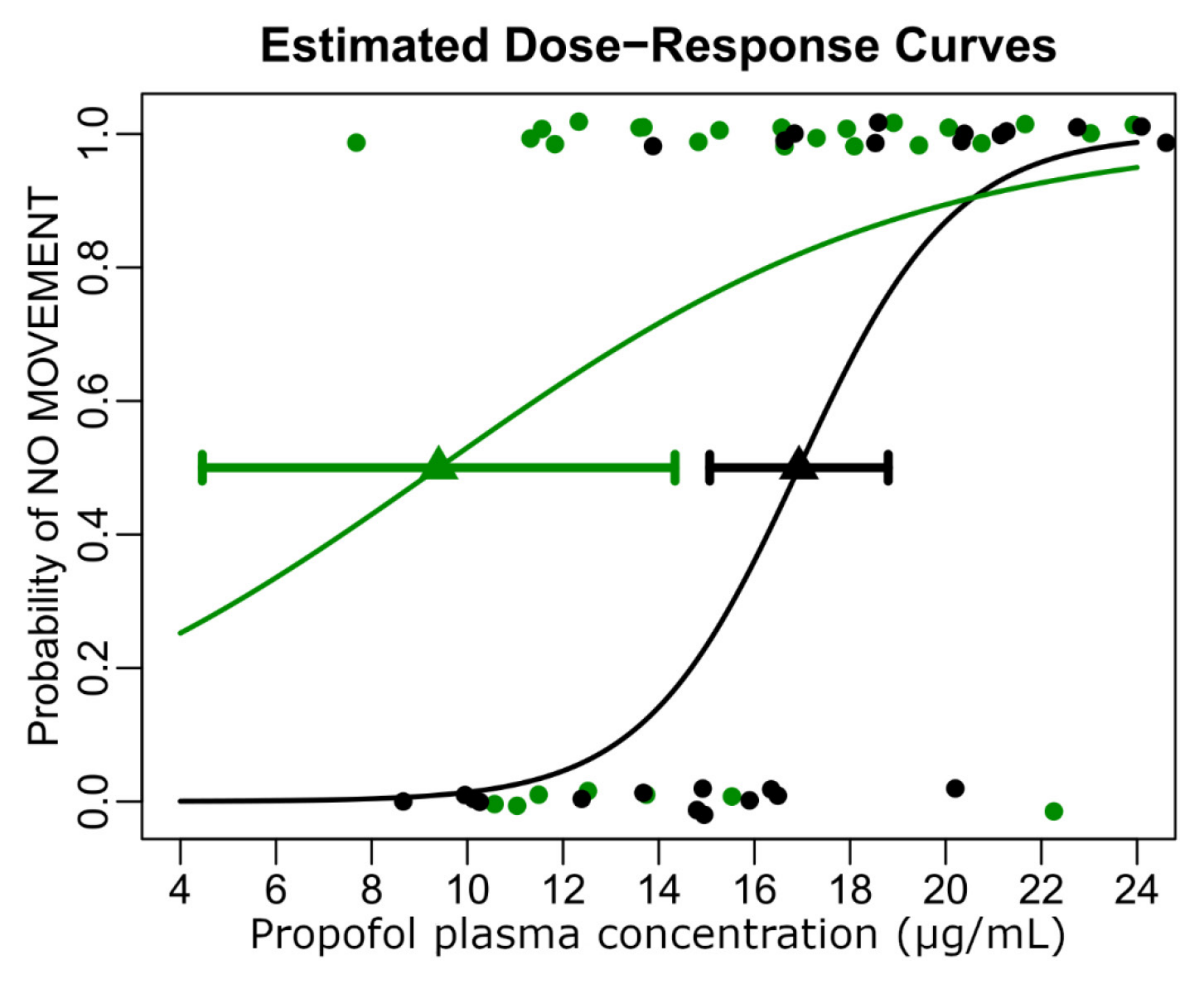
Estimated dose–response curves for the placebo (black line) and the pregabalin (green line) groups using logistic regression based on measured propofol plasma concentrations. The Cp50 values of propofol for both groups are shown as triangles, with the corresponding 95% confidence intervals (calculated using the delta method) shown as horizontal lines. The x-axis represents the propofol plasma concentration in μg/mL. Individual observations are shown as jittered points at 1 (no movement) and 0 (movement) along the x-axis at each concentration. Jittering was added to visually represent the number and distribution of binary responses and does not indicate values other than 0 or 1.

The difference between the target effect-site and plasma propofol concentration was compared across both groups using paired *t* tests at 15 and 20 minutes after the start of the infusion. To evaluate the associations between set and measured concentration in more detail, Spearman correlation coefficients were calculated. Furthermore, subject characteristics, secondary outcome parameters, and side effects were compared between groups. Continuous parameters were visually checked for normal distribution, outliers, or skewness using histograms and q–q plots. Parameters were then compared between groups using *t* tests or the Mann–Whitney *U* test. Categorical variables, such as ASA physical status, were analyzed using the χ^2^ test. Due to the lower number of events, Fisher exact test was used for binary outcomes, including awareness, nausea, vomiting, dizziness, visual impairment, other side effects, and the administration of ondansetron or metamizole. The significance level was set to 0.05. All analyses were performed using R, release 4.2.2.

## RESULTS

### Cp50 of Propofol Target Concentration

In total, 88 patients were recruited for this study. Eight patients were excluded after randomization and before receiving the intervention due to medical or organizational reasons (Figure 1). These participants were replaced to maintain the planned sample size, and analysis was conducted per protocol. There was no missing data among participants included in the final analysis. All patients who received the intervention completed the study protocol, and all relevant outcome data were collected. The Cp50 was estimated to be 16.9 µg/mL in the placebo group and 9.4 µg/mL in the pregabalin group, using the delta method and based on measured propofol plasma concentrations taken 20 minutes after induction, immediately before skin incision. The results indicate a statistically significant difference according to the delta method, as the CI for the difference did not include 0. Validation through the bootstrap method revealed similar results, although its CIs showed some overlap as shown in Table 2. The logistic regression models estimated a dose–response curve with an intercept of −10.4 (*b*0) and a slope of 0.62 (*b*1) in the placebo and with an intercept of −1.89 (*b*0) and a slope of 0.20 (*b*1) in the pregabalin group (Figure 2). The number of gross purposeful movements at different propofol target concentrations, separately for the placebo and pregabalin groups, is provided in the digital Supplemental Digital Content, Supplementary Table, https://links.lww.com/AA/F571.

### Reasons for Exclusion After Randomization

Six patients were excluded after randomization for medical reasons. The specific reasons for exclusion included one patient’s inability to swallow the study medication, impossible LMA that required intubation (2 patients), impossible arterial line after 3 attempts by 2 different physicians (2 patients), and fever on the day of surgery (1 patient). Two patients were excluded for organizational reasons because of acute changes in the surgical schedule, resulting in a significantly shorter time span between applying pregabalin and the surgical procedure.

### Propofol and Pregabalin Plasma Concentrations

Pregabalin plasma concentrations were measured immediately before the skin incision. Pregabalin was not detected in the placebo group. In the pregabalin group, the mean plasma concentration was 7.67 μg/mL. Propofol plasma concentrations were measured 15 and 20 minutes after the start of propofol infusion.

The difference between the target effect-site and plasma propofol concentrations at 15 and 20 minutes after the start of the infusion was statistically significant across all target concentrations (paired *t* test *P* values <.001 for each concentration level). Specifically, for each predefined effect-site target concentration, the corresponding mean measured plasma concentration was compared to the target effect-site concentration using paired *t* tests. This comparison was performed separately for each group and at both time points, as shown in Table 3. Additionally, Spearman’s correlation coefficient between the target effect-site concentration and the absolute difference between plasma and target effect-site concentrations was 0.859 (*P* < .001). The correlation between the target effect-site concentration and the ratio of plasma to target effect-site concentrations was also statistically significant (*r* = 0.526, *P* < .001).

**Table 3.** Propofol Target Effect-Site Concentrations and Plasma Concentrations After 15 and 20 min

Target effect-site concentration	n	Plasma 15 min	Plasma 20 min	Difference	Ratio
5.5	11	10 (2.11)	10.6 (1.63)	5.12 (1.63)	1.93 (0.3)
7	13	13.9 (2.37)	14.8 (2.79)	7.78 (2.79)	2.11 (0.4)
8.5	14	16.0 (2.62)	17.1 (2.12)	8.56 (2.12)	2.01 (0.25)
10	13	20.0 (2.28)	21.4 (3.32)	11.4 (3.32)	2.14 (0.33)
11.5	12	26.6 (4.76)	27.2 (4.31)	15.7 (4.31)	2.36 (0.37)
13	9	29.9 (5.4)	31.0 (3.69)	18.0 (3.69)	2.39 (0.28)
14.5	8	34.1 (4.64)	38.6 (4.29)	24.1 (4.29)	2.66 (0.3)

Propofol target effect-site concentrations and plasma concentrations after 15 and 20 min. Differences between target effect-site concentrations and plasma concentrations after 20 min (μg/mL) and ratios between target effect-site concentrations and plasma concentrations after 20 min. Data are expressed as means and standard deviations and are rounded to three significant digits.

**Table 4.** Cp50 of Propofol Target Effect-Site Concentrations

Cp50 of propofol target effect-site concentrations		
Group	Delta method	Bootstrap
Placebo	8.47 (7.55–9.39)	8.46 (7.51–9.37)
Pregabalin	5.62 (3.71–7.52)	5.49 (2.81–6.89)
Difference	2.85 (0.87–4.84)	2.97 (1.18–6.05)

Cp50 (µg/mL) and corresponding 95% confidence limits separately for the target propofol effect-site concentrations in the placebo and the pregabalin group, as well as for the difference between groups. Data are rounded to three significant digits.

**Figure 3.**
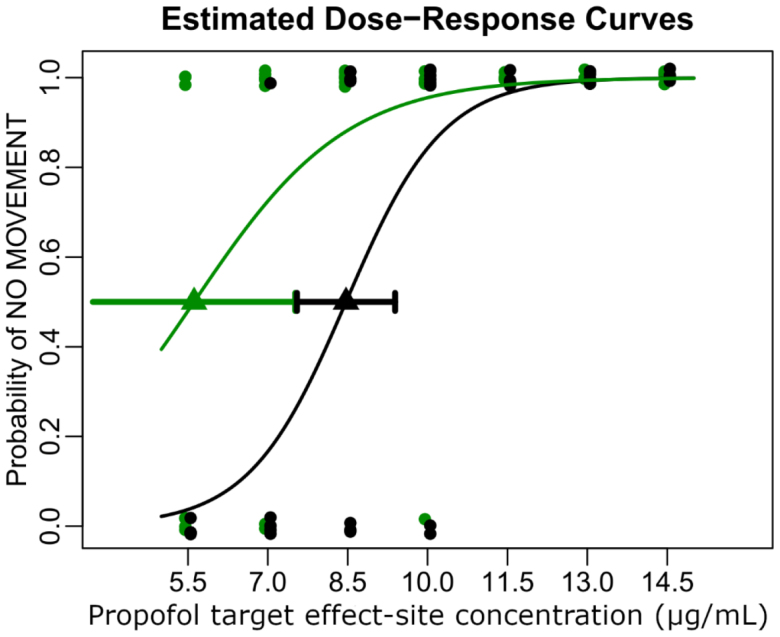
Estimated dose–response curves for the placebo (black line) and the pregabalin (green line) groups using logistic regression based on propofol target effect-site concentrations. The Cp50 values of propofol for both groups are shown as triangles, with the corresponding 95% confidence intervals (calculated using the delta method) shown as horizontal lines. The x-axis represents the propofol target effect-site concentration in μg/mL. Individual observations are shown as jittered points at 1 (no movement) and 0 (movement) along the x-axis at each concentration. Jittering was added to visually represent the number and distribution of binary responses and does not indicate values other than 0 or 1.

For comparison, statistical analyses of Cp50 were also performed using the target effect-site concentrations. In this case, the Cp50 of propofol was estimated to be 8.47 μg/mL in the placebo group and 5.62 μg/mL in the pregabalin group (Figure 3). This difference was statistically significant based on both the delta and bootstrap methods, as shown in Table 4.

### Postoperative Pain Levels and Opioid Consumption

Patients received piritramide bolus dosages of up to 4.5 mg for pain management using the PCA approach. Two hours after the procedure, the patients were asked to rank their pain levels on an NRS ranging from 0 (no pain) to 10 (worst pain possible). In the placebo group, 27 patients asked for analgesics; in the pregabalin group, 24 patients asked for it. The mean dose of piritramide was 4.51 (standard deviation [SD] = 3.91) mg in the placebo group and 2.52 (SD = 2.64) mg in the pregabalin group. The mean NRS in the placebo group was 3.1 (SD = 1.77) and 1.7 (SD = 1.77) in the pregabalin group, indicating a statistically significant difference in pain levels 2 hours postoperatively (Table 1).

### Secondary Outcome Parameters

There were no differences in the overall satisfaction or satisfaction with the induction of anesthesia. None of the patients showed signs of intraoperative awareness or discomfort during the induction of anesthesia. In the postanesthesia care unit (PACU), 3 patients in the placebo group and 6 patients in the pregabalin group reported dizziness, yet the difference was not statistically significant (*P* = 0.481). In both groups, 1 patient reported nausea. No other side effects, such as vomiting or visual impairment, were detected. No statistically significant differences were found between the 2 groups regarding BIS, heart rate, or blood pressure before or after skin incision. A significantly higher heart rate before skin incision was observed in the placebo group (M = 71, SD = 11.0) compared to the pregabalin group (M = 66, SD = 11.3; *P* = .043). Furthermore, a statistically significant difference in BMI between the placebo group (M = 23.2, SD = 2.58) and the pregabalin group (M = 24.8, SD = 3.00) was observed (*P* = .015). However, this difference would not remain significant after correcting for multiple comparisons, such as with a Bonferroni adjustment. This likely reflects random variation, as the groups were randomized but not stratified by BMI.

## DISCUSSION

This study found that in female patients undergoing breast surgery, pretreatment with 300 mg pregabalin reduced the Cp50 of propofol by 44%, as calculated from measured plasma concentrations immediately before skin incision. Based on the Schnider algorithm for target effect-site concentrations, the Cp50 was reduced by 34%.

This pain-modulating effect of pregabalin can be attributed to its mechanisms in experimental models. Pregabalin functions as a calcium channel antagonist, binding with high affinity to the α2δ1 subunit, a specific subunit that mediates hypersensitivity and modulates dorsal horn responses to peripheral pain stimuli. Furthermore, pregabalin inhibits proinflammatory cytokines and activates the descending inhibitory system, exerting analgesic effects.^14–16^ In clinical settings, pregabalin has also been demonstrated to reduce the MAC of sevoflurane.^4^

However, conflicting results have been reported regarding the effects of pregabalin on propofol anesthesia requirements. One experimental study found that pregabalin premedication reduced the amount of propofol required to achieve specific BIS values.^7^ Conversely, other authors reported that pregabalin does not reduce the effective dose (ED50) necessary to achieve predetermined entropy-monitoring values.^6^ Notably, there are several methodological differences between these studies and ours.

First, we tested only propofol and pregabalin, whereas Moreau-Bussière et al’s^6^ study included coadministration of lidocaine to reduce injection pain. Lidocaine itself has been shown to reduce the Cp50 of propofol,^5^ and this effect may, therefore, have influenced their result.

Second, we did not use electroencephalogram (EEG)-based variables such as BIS as outcome parameters but measured the motor response to skin incision. While the EEG is recorded from the brain, the motor response to skin incision is believed to be mainly located in the spinal cord.^17^ Propofol acts primarily on the brain,^18^ whereas volatile anesthetic agents, such as sevoflurane, also act on the spinal cord.^19^ This might explain why comparatively high doses of propofol are required to suppress the motor response to skin incision when not combined with opioids or other anesthetic agents. As shown in Table 1, the BIS values in our study were exceptionally low, which is expected at high propofol dosages. In clinical practice, opioids are administered during surgery, resulting in a significant reduction in the amount of propofol required for adequate anesthesia. Given that opioids have a limited effect on BIS values, higher BIS values are observed during routine general anesthesia.^20^

We also investigated postoperative pain levels and opioid consumption as secondary outcome parameters. Opioid requirements in the recovery room were significantly lower in the group that received 300 mg pregabalin compared to the placebo group. Additionally, at 2 hours postoperatively, the mean NRS score was significantly lower, indicating a stronger opioid effect. Other studies reported similar opioid-sparing effects.^21,22^

In this study, we used a randomized method to determine the Cp50 of propofol, whereas similar studies used up-and-down titration methods to estimate the Cp50 of various anesthetic agents.^4,5,7,23,24^ The main advantage of the up-and-down method is that it requires fewer subjects, as the target concentrations vary less than with other methods. However, its major drawback is the requirement for target concentration to match the effect-site concentration for reliability. To the best of our knowledge, no reliable method currently exists to measure or estimate the propofol effect-site concentration in real-time, making the up-and-down method unsuitable for estimating the Cp50 of propofol.

We measured plasma levels twice in our patients, and while there were slight variances in the high dosage groups between 15 and 20 minutes (Table 3), plasma levels appeared stable after 20 minutes. Significant differences were observed between the measured propofol plasma concentrations and propofol target effect-site concentrations. For measured propofol plasma concentrations, we estimated the Cp50 in the placebo group to be 16.9 µg/mL (Table 4), aligning with Smith et al,^25^ who found the Cp50 for skin incisions to be 15.2 µg/mL and Andrews et al,^10^ who found it to be 14.3 µg/mL. The infusion pump was regularly maintained by the manufacturer and the in-house service department, and we also benchmarked it against models from different manufacturers.

The Schnider model is known to underestimate propofol plasma concentrations.^26–28^ Most studies report up to 30% discrepancies when testing target effect-site concentrations up to 5 µg/mL.^26–28^ There seems to be less scientific interest in higher concentrations, as no studies evaluate the performance of the Schnider model in target effect-site concentrations >10 µg/mL. This lower dosage range is reasonable for routine clinical scenarios in which propofol is combined with opioids or other anesthetic agents. However, higher concentrations were needed in our study since propofol was the sole anesthetic. We noticed higher deviations toward the upper end of the tested concentrations, whereas, at the lower end, deviations were less pronounced, aligning with other studies reporting dose-dependent inaccuracies.^27^

One methodological limitation of this study is the limited resolution at the lowest administered propofol target effect-site concentration (5.5 μg/mL), particularly in the pregabalin group, where the Cp50 lies near this lower boundary. For safety and blinding reasons, lower concentrations were not administered. While this approach allowed for greater precision near the expected Cp50 by allocating more patients to mid-range concentrations, it may have reduced statistical power for estimating the lower end of the dose–response curve. This was especially relevant in the bootstrap analysis based on measured propofol plasma concentrations, which did not confirm statistical significance. However, the delta method did confirm a statistically significant Cp50 reduction in this analysis, and both methods confirmed statistical significance when based on the propofol target effect-site concentrations, supporting the robustness of the overall findings. Our results also suggest that the initially anticipated safety concerns associated with lower propofol concentrations (particularly in the pregabalin group) may be less critical than expected. Future studies should consider including lower concentrations in this group to more precisely characterize the dose–response relationship in this range.

Another limitation of our study is that we did not measure opioid consumption and pain levels after the patients were transferred out of the recovery room, where pregabalin’s effect might have diminished. If the coanalgesic properties and side effects of pregabalin were to be determined in the general ward or across the entire hospital stay, a repetitive regimen of pregabalin should be in place, as the half-life of pregabalin is approximately 6 hours.^29^

Only female patients were recruited for the study due to the high standardization of breast surgery and consistent patient characteristics, like ASA score and physical fitness, compared to other types of surgeries. Although the Schnider algorithm accounts for the patient’s sex, we cannot entirely exclude that pregabalin’s effect on propofol’s Cp50 might be sex-related. However, this seems unlikely because no sex-specific effects of pregabalin have been described.

In conclusion, preoperative administration of 300 mg pregabalin reduced the Cp50 of propofol by 44% when calculated using measured plasma concentrations immediately before skin incision, and by 34% when using effect-site concentrations as estimated by the Schnider pharmacokinetic model. Statistically significant differences were observed between propofol plasma levels and corresponding target effect-site concentrations. Additionally, pregabalin may reduce postoperative pain and opioid consumption.

## Acknowledgments

The authors wish to thank Dr. Thomas Hamp and Dr. Edith Fleischmann for their roles as scientific advisors. We also thank Dr. Fleischmann for providing care for study patients and facilitating administrative procedures. Our gratitude extends to the breast surgeons and gynecologists who facilitated study procedures, particularly Dr. Daphne Gschwantler-Kaulich, Dr. Christine Deutschmann, Dr. Carmen Leser, and Dr. Georg Pfeiler.

## DISCLOSURES

**Conflicts of Interest:** None. **Funding:** None. **This manuscript was handled by:** Jiro Kurata, MD, PhD.

## Supplementary Material

## Footnotes

a LMA^®^ Supreme Airway™ is a registered trademark of Teleflex Incorporated or its affiliates.
