# Supplementary material for: Preoperative Pregabalin and the Cp50 for Skin Incision During Target-Controlled Propofol Infusion: A Randomized, Placebo-Controlled, Double-Blind Clinical Trial
Source: Anesth Analg. 2025 Oct 29;143(2):373–82. doi: 10.1213/ANE.0000000000007824 (PMC13326925; doi:10.1213/ANE.0000000000007824)
Supplement: Supplementary file 1 [file ane-143-373-s001.pdf]

Supplementary Table: Number of gross purposeful movements separately for different propofol target concentrations in the placebo and the pregabalin group

	Placebo			Pregabalin		
**Target effect-site concentration**	**n**	**Movement**	**No movement**	**n**	**Movement**	**No movement**
**5.5**	4	4	0	7	4	3
**7.0**	5	4	1	8	2	6
**8.5**	7	3	4	7	0	7
**10.0**	8	2	6	5	1	4
**11.5**	7	0	7	5	0	5
**13.0**	5	0	5	4	0	4
**14.5**	4	0	4	4	0	4
